# Knowledge and attitudes about health research amongst a group of Pakistani medical students

**DOI:** 10.1186/1472-6920-6-54

**Published:** 2006-11-02

**Authors:** Hassan Khan, Muhammad RizwanulHaq Khawaja, Abdul Waheed, Muhammad Ameen Rauf, Zafar Fatmi

**Affiliations:** 1 Medical College, Aga Khan University, Karachi, Pakistan; 2 Department of Community Health Sciences, Aga Khan University, Karachi, Pakistan

## Abstract

**Background:**

Health research training is an important part of medical education. This study was conducted to assess the level of knowledge and attitudes regarding health research in a group of Pakistani medical students at Aga Khan University, Karachi.

**Methods:**

It was a cross-sectional pilot study conducted among a group of Pakistani medical students. Through stratified random sampling, a pre-tested, structured and validated questionnaire was administered to 220 medical students. Knowledge and attitudes were recorded on a scale (graduated in percentages).

**Results:**

Mean scores of students were 49.0% on knowledge scale and 53.7% on attitude scale. Both knowledge and attitudes improved significantly with increasing years of study in medical college [Regression coefficient 4.10 (p-value; 0.019) and 6.67 (p-value; < 0.001) for knowledge and attitudes, respectively].

**Conclusion:**

Medical students demonstrate moderate level of knowledge and attitude towards health research. Intensive training in this regard is associated with significant improvement in knowledge and attitudes of students towards health research.

## Background

Health research training forms an important part of medical education [1]. It is essential to inculcate critical thinking and reasoning skills and to develop a positive attitude amongst students towards scientific research from the beginning of their medical career [2]. Studies have proven that involvement in research as a medical student is strongly associated with postgraduate research initiatives [3,4].

Unfortunately, the number of physician scientists has declined over the past two decades and there is a dire need for more clinical as well as basic health science investigators. The role of undergraduate research assistants is thus ever more important [5,6]. Encouraging and motivating students' research activity can fill up the void of physician scientists and help developing countries to achieve self-reliance in health care and research [1].

The new trends in medical education focus on acquisition of skills, knowledge and attitudes rather than factual learning [7]. New and innovative methods are being employed which stress on development of lifelong self directed learning skills. The problem based learning (PBL) approach as an educational strategy is gaining popularity amongst medical institutions all over the world [8]. Therefore, contribution of newer approaches, like PBL, towards improving the knowledge and attitudes of students about research also need to be evaluated.

With this background, we assessed the attitudes and knowledge of medical students of Aga Khan University towards medical research as a direct indicator of their understanding and acceptance of new findings having potential to influence health care. We also investigated factors such as gender, age, type of high school course and year of study at medical school involved in influencing students' knowledge and interest in scientific research.

## Methods

### Study design and study site

This cross sectional, knowledge and attitude survey was conducted among medical students of Aga Khan University (AKU); a private educational institution in Karachi, Pakistan. Maintaining a tertiary care health facility, the university attracts medical students from all parts of the country.

In Pakistan, medical schools offer a 5 years programme leading to an MB; BS (Bachelors of Medicine; Bachelors of Surgery) degree. Basic health sciences are the primary focus of instruction during the first two years, with gradually increasing exposure to clinical rotations over the next three years. Aga Khan University has recently introduced the problem based learning (PBL) curriculum. At the time of this study three classes (year 1–3) were following problem based learning (PBL) mode courses, while year 4 and 5 were being taught through conventional lecture based learning (LBL).

### Study sampling

At the time of study, a total of 420 students were enrolled at the medical college. We required a sample size of 220 subjects to fulfill the objectives of our study at a 95% confidence level. This sample size was calculated assuming a 50% prevalence of good knowledge and attitude, 5% bond-on error, and 10% non-response rate. The students were randomly chosen according to the probability of proportionate sizes of the classes – stratified random sampling.

The questionnaires were distributed after seeking verbal consent and students were requested to return them within two days. One hundred and ninety-seven students (response rate of 89.5%) returned the completed form and were included in the analysis.

### Questionnaire

A pre-tested, structured questionnaire was adapted with permission from questionnaire validated by Vodopivec *et al *[8]. For adaptation of the questionnaire a thorough peer review and discussions was done. The questionnaire was then pre-tested on a group of students who were expected to identify questions most valid in ascertaining our objectives. These were accordingly modified to develop a final questionnaire.

The questionnaire consisted of three parts namely; student's profile, evaluation of student's knowledge and attitudes of health research. Demographic details of subjects included age, gender, type of high school course, year of study and mode of learning at medical school (PBL versus LBL) and place of origin. The high schools were classified into Pakistani Higher Secondary School Certificate (HSSC), British Advanced Levels (A-levels) and Others. Mode of learning was classified into conventional lecture based learning (LBL) and problem based learning (PBL). Knowledge was assessed by ten multiple-choice questions. For each student, the percentage of correct answers was calculated as a representative of knowledge score. Six questions were asked to assess the attitudes of students towards health research and each answer was scored on a scale of 0.0 (unfavorable attitude) to 1.0 (favorable attitude). For each individual, score of individual questions was summed and then converted into percentage to represent the attitude score.

### Statistical analysis

Data was entered and analyzed in Statistical Package for Social Sciences 13.0 (SPSS, Inc., Chicago, IL, USA). Descriptive statistics were performed for mean scores and proportions. Multiple linear regression model was used to test association of age and year of study with the knowledge and attitude. ANOVA and t-test were used to look for similar putative associations of type of high school, mode of study and gender. Results were recorded as frequencies, means ± standard deviations (SD), p-values, standardized and unstandardized regression coefficients. For all purposes, a p-value of <0.05 was considered as the criteria of significance.

## Results

Of 197 students, 122 (62.6%) were males and 73 (37.4%) females. Mean age of the study participants was 20.92+1.79 years. Mean percentage score (SD) of the population was 49.0% (19.7) on knowledge scale and 53.7% (21.4) on attitude scale. Table 1 shows the proportion of students in quartiles of knowledge and attitude scores.

Table 2 shows the number of students in different groups with respect to gender, type of high school, years of education and mode of learning at medical school. Mean scores [± Standard Deviation] on a knowledge and attitude scale were also compared. Males scored better on the attitude scale, though the difference on the scale of knowledge was not significant. Type of high school course was not a significant predictor of knowledge or attitude about research. Conventional LBL was associated with a better score on both knowledge and attitude scales.

Table 3 shows the predictors of score on knowledge and attitude scale through a multivariate linear regression model. Age was not a statistically significant factor in determining scores on both knowledge and attitude scales. After adjusting for age, the number of years spent at medical school was a significant predictor of both knowledge and attitude scores. Increase in duration of study by one year increased the knowledge score by 4.1% with a correlation coefficient (r) of 0.30 and coefficient of determination (r^2^) of 0.09 [p-value = 0.019]. Similarly, increase in duration of study by one year increased the attitude score by 6.67% with a correlation coefficient (r) of 0.45 and coefficient of determination (r^2^) of 0.20 [p-value < 0.001].

The proportion of subjects with correct answer for each knowledge question has been shown in Table 4. Table 5 demonstrates different responses for each attitude question. Out of 178 (90.3%) students feeling confident in interpreting and writing a research paper, 56 (28.4%) could do it without assistance, while 122 (61.9%) preferred to do so with assistance.

## Discussion

This study reports moderate level of knowledge towards health research [mean score 49%] among Pakistani medical students. About 80% of the students were falling in the middle two quartiles of the knowledge score, second quartile accounting for more than 50% of the population. Similar trends were demonstrated by the students on the attitude score [mean score = 53%]. Our findings are comparable with the results of Vodopivec *et al*., who conducted a study with similar questionnaire among first year Croatian medical students [8]. Comparing the results of first year medical students of AKU and Croatia yields a similar mean knowledge (43.2% vs. 44%), but a lower mean attitude (39.2% vs. 62.5%) score. However, this score better represents the baseline affect of secondary and high school education on knowledge and attitudes for research of students. About 90% of AKU medical students felt confident in interpreting and writing a research paper. Nevertheless, only 28.4% claimed the ability to do so without needing any assistance.

Students' knowledge and attitude towards health research significantly improved with increasing years of education at medical school. This signifies a relatively satisfactory contribution of medical curriculum in developing research skills among medical students through well-structured intensive training. Students at AKU are taught theoretical essentials of research methodology, statistics and epidemiology during the first two years of their medical curriculum. This is followed by extensive community health projects undertaken by groups of students during year 4 and 5. During these projects, students are involved in designing and implementing their research questions, analyzing their data and writing a detailed report of their project. Several of these studies get published in indexed journals. Mandatory participation in research activity has been shown to improve students' knowledge and attitudes towards research [9].

A recent audit of the students' corner of the Journal of Pakistan Medical Association revealed that more than 75% of publications were contributed by AKU students [10]. Students contributing to local research addressing specific health problems of the local community can have important implications in influencing the clinical practices in that area [11,12]. The results are also encouraging in the context of a third world country with relatively poor research involvement of physicians of earlier generations [2]. Moreover, research experience as a medical student is strongly associated with future research involvement [10].

Gender was not a significant predictor of knowledge about health research. However, males had a significantly higher mean score on the attitude scale. Students' high school category did not affect their knowledge or attitude scores. Majority of the students in our study were involved in research projects other than the mandatory course work in community projects. Moreover, most of them felt that medical students could carry out their independent research.

Our study showed a significant difference between knowledge and attitude scores of conventional LBL (year 4 and 5) students and PBL (year 1 to 3) students. However, these scores also increased with higher year of study. Therefore, the role of 'year of study' as a confounder between mode of study and knowledge and attitude scores cannot be ruled out. It might be leading to a spuriously significant difference in the knowledge and attitudes of PBL and LBL students. Several studies have established that there is no significant difference between the knowledge and competency of PBL and conventionally trained students [13]. However, whether the students in these two groups differ with respect to their approach towards research needs to be further investigated.

The study was conducted at one institution to serve as a pilot for a large scale research. So the findings cannot be generalized for the whole population of Pakistani medical students. In spite of these limitations, the use of a validated questionnaire allows us to compare our findings to other studies done under similar settings and using the same evaluative tool. We recommend further detailed studies to be carried out across health institutes all over the country to address this critical issue of research. Furthermore other factors influencing research activity such as funding, research infrastructure, rising cost of medical education, student debts and adequate research opportunities need to be evaluated.

## Conclusion

In conclusion, we report moderate knowledge and attitude among a group of Pakistani medical students about the health research at the beginning of medical school. However, significant improvements were seen over the years owing to training and research involvement. It is still unclear whether there is a difference between LBL and PBL students in their knowledge and attitudes towards health research. The need for health researchers will increase in the future context of increasing burden of communicable and non-communicable diseases in the developing countries [14]. Therefore, we must invest in developing a medical curriculum that is superior and more robust in catering the health research demands of the society.

## Competing interests

The author(s) declare that they have no competing interests.

## Authors' contributions

HK and AW conceived and designed the study. HK, AW and MAR administered questionnaires. MRK, MAR and ZF managed, analyzed and interpreted data. HK, MRK and ZF prepared the manuscript. All authors approved the final manuscript.

## Pre-publication history

The pre-publication history for this paper can be accessed here:



## Figures and Tables

**Table 1:** Proportion of Pakistani Medical Students Falling in Different Score Categories

Score Category	Knowledge	Attitude
	
	Students %	Students %
<25	11.7	8.2
26–50	51.3	42.3
51–75	27.9	38.8
>76	9.1	10.7

**Table 2:** Pakistani Medical Students' Knowledge and Attitude towards Research According to Gender, Type of High School, Year and Mode of Learning at Medical School

		No.	Knowledge		Attitude	
			
			Mean+SD	p-value	Mean+SD	p-value
Gender	Male	122	49.4+19.8	0.705	57.7+21.7	0.001
	Female	73	48.4+18.6		47.2+19.1	
High school type	HSSC	73	47.5+20.9	0.349	54.2+18.9	0.615
	A-Levels	110	50.0+18.5		53.4+22.4	
	Others	12	55.8+16.2		59.7+20.7	

Mode of learning	PBL	131	45.7+20.9	<0.001	49.0+18.6	<0.001
	LBL	66	55.5+15.3		63.5+23.1	

Year at medical school	1st	47	43.2+19.0		39.2+16.0	
	2nd	46	47.0+21.5		55.4+18.1	
	3^rd^	38	47.4+22.7		53.7+17.3	
	4^th^	32	58.4+17.3		60.7+27.2	
	5th	34	52.6+12.9		66.2+18.6	

Total		197	49.0+19.7		53.7+21.4	

HSSC, Higher secondary school certificate; A-levels, Advanced level; PBL, Problem based learning; LBL, Lecture based learning; SD, Standard deviating

**Table 3:** Predictors of Score on the Knowledge and Attitude Scales among Pakistani Medical Students

	Regression Coefficient (b)	Correlation coefficient (r)	p-value
Knowledge			

Year at medical school	4.1	0.30	0.019
Age	-0.98	-0.90	0.476

Attitude			

Year at medical school	6.67	0.45	<0.001
Age	-0.63	-0.05	0.661

**Table 4:** Proportion of Pakistani Medical Students with Correct Answers for Questions¶

	No. (%)
1. How would you define the scientific hypothesis?	58 (29.4)
a. A proposed idea or thought	
b. An answer or solution to a question	
c. An answer or solution to a question which has a capacity of verification or empirical demonstration*	
d. logical deduction of the premises that may or may not be verified empirically	
	
2. How would you define scientific theory?	89 (45.2)
a. Speculation or assumption with no or insufficient evidence	
b. Scientific hypotheses that may be proven, but lacking evidence for verification.	
c. Set of scientific knowledge on a given topic or area	
d. System of hypotheses logically connected to one another, with common background, some of which have been verified*	
	
3. How would you define the scientific truth?	135 (68.5)
a. The truth that will be reached through scientific research	
b. Absolute truth	
c. Consensus of competent experts *	
d. Fact that can be found in the textbooks	
e. Facts that your professors teach you	
	
4. The essential characteristic of science is:	47 (23.9)
a. All scientific conclusions are temporary*	
b. Scientific theory cannot merely explain natural phenomena, but must somehow also exert influence upon them	
c. Rather obvious scientific conclusion does not have to be testable	
d. An experiment is not an objective model of the nature but serves as an introduction into real research of natural phenomena	
	
5. A scale from 1 to 5 (like grades on an examination) is called:	96(48.7)
a. Ratio scale	
b. Nominal	
c. Ordinal *	
d. Interval	
e. It is not a scale	
	
6. Representativeness is a key characteristic of a:	106 (53.8)
a. Scientific paper	
b. Professional paper	
c. Scientific research	
d. Sample*	
e. Population	
	
7. MEDLINE is:	120 (60.9)
a. The first and best known "on-line" medical journal	
b. International association of medical informaticians	
c. Printed form of the Excerpta Medica	
d. Abbreviation (acronym) that lists the parts of the research article	
e. Medical database*	
	
8. In the previous year, you have published a paper in a prestigious Journal of Immunology. Now you want to check the number of citations your paper has received. The best way to do it would be to search the:	81 (41.1)
a. author index of the MEDLINE database	
b. Corporate index of the Science Citation Index database	
c. Author index of the Current Contents database	
d. Citation index of the Science Citation Index database*	
e. Author index of the Science Citation Index database	
	
9. The part of a scientific paper is:	119 (60.4)
a. Author's curriculum vitae	
b. Letter to the editor enclosed with the paper	
c. Description of the timeline	
d. Acknowledgment to persons who assisted you during the research*	
	
10. All listed rules apply to the process of writing an Introduction section of a scientific paper EXCEPT:	114 (57.8)
a. clearly state why the research has been started	
b. do not explain textbook facts	
c. do not explain words from the title of the paper	
d. make it longer rather than shorter*	
e. clearly define the question to which your research aims to provide an answer	

Mean score (+ SD)	49.0 + 19.7

¶Questions used with permission of Vodopivec *et al. *[8], *Correct

**Table 5:** Responses to Questions Determining Attitudes of Pakistani Medical Students Towards Scientific Research

Statement	Yes	No	Undecided
1. Do you feel confident in interpreting and writing a research paper?	178 (90.3%)	17 (8.6%)	2 (1.0%)
2. Have you ever participated in a research project (apart from mandatory academic projects)?	103 (52.3%)	92 (46.7%)	2 (1.0%)
3. Have you ever written a scientific paper?	51 (25.9%)	145 (73.6%)	1 (0.5%)
4. Do you think undergraduate students should participate in research?	180 (91.4%)	16 (8.1%)	1 (0.5%)
5. Do you think undergraduate students can plan and conduct a research project and write a scientific paper?	171 (86.8%)	21 (10.7%)	5 (2.5%)
6. Medical students can plan and conduct research project without supervision	120 (60.9%)	51 (25.9%)	26 (13.2%)
